# Spatiotemporal heterogeneity and impact factors of hepatitis B and C in China from 2010 to 2018: Bayesian space–time hierarchy model

**DOI:** 10.3389/fcimb.2023.1115087

**Published:** 2023-02-27

**Authors:** Jiaojiao Qian, Ming Yue, Peng Huang, Lele Ai, Changqiang Zhu, Chongcai Wang, Yizhe Luo, Na Yue, Yifan Wu, Yun Zhang, Chunhui Wang, Weilong Tan

**Affiliations:** ^1^ Department of Epidemiology, School of Public Health, Nanjing Medical University., Nanjing, China; ^2^ Department of infectious diseases prevention, Nanjing Bioengineering (Gene) Technology Center for Medicines, Nanjing, China; ^3^ Department of Infectious Diseases, the First Affiliated Hospital of Nanjing Medical University, Nanjing, China; ^4^ Department of infectious diseases prevention, Hainan International Travel Healthcare Center, Haikou, China

**Keywords:** hepatitis B, hepatitis C, Bayesian model, spatiotemporal heterogeneity, GDPta, illiteracy rate

## Abstract

**Introduction:**

Viral hepatitis is a global public health problem, and China still faces great challenges to achieve the WHO goal of eliminating hepatitis.

**Methods:**

This study focused on hepatitis B and C, aiming to explore the long-term spatiotemporal heterogeneity of hepatitis B and C incidence in China from 2010 to 2018 and quantify the impact of socioeconomic factors on their risk through Bayesian spatiotemporal hierarchical model.

**Results:**

The results showed that the risk of hepatitis B and C had significant spatial and temporal heterogeneity. The risk of hepatitis B showed a slow downward trend, and the high-risk provinces were mainly distributed in the southeast and northwest regions, while the risk of hepatitis C had a clear growth trend, and the high-risk provinces were mainly distributed in the northern region. In addition, for hepatitis B, illiteracy and hepatitis C prevalence were the main contributing factors, while GDP per capita, illiteracy rate and hepatitis B prevalence were the main contributing factors to hepatitis C.

**Disussion:**

This study analyzed the spatial and temporal heterogeneity of hepatitis B and C and their contributing factors, which can serve as a basis for monitoring efforts. Meanwhile, the data provided by this study will contribute to the effective allocation of resources to eliminate viral hepatitis and the design of interventions at the provincial level.

## 1 Introduction

In 2015, the global hepatitis virus caused 10 million new infections and 1.3 million deaths, of which 96% were caused by chronic infection caused by hepatitis B virus (hepatitis B) and hepatitis C virus (hepatitis C). Hepatitis B virus and hepatitis C virus have parallel transmission routes, so a certain proportion of patients can have dual virus infection (Raimondo and Saitta, 2008; Brass and Moradpour, 2009; Riaz et al., 2011). Patients with co infection have a 2-3 fold increased risk of advanced liver disease (Liu et al., 2014). In 2016, the World Health Organization called on the world to fight against viral hepatitis and eliminate hepatitis by 2030 through expanded prevention, detection and treatment(Organization W.H., 2016). Eliminating hepatitis is defined as reducing the incidence rate by 90% and mortality by 65% on the basis of 2015. Studies(2017; WHO, 2016; Ward and Hinman, 2019) have shown that eliminating viral hepatitis was feasible because of the characteristics of HBV and HCV, the availability of HBV vaccines and other interventions to prevent transmission, reliable diagnosis, and drugs to treat HBV and cure HCV before the onset of serious disease and premature death. The burden of hepatitis B and C in China is enormous, with an estimated 70 million hepatitis B surface antigen (HBsAg) carriers (prevalence 5%-6%) in China (Liu et al., 2019), while the prevalence of hepatitis C is estimated at 1.3% (Gower et al., 2014). Although China has invested heavily in basic research, vaccine and drug development, and mandated hepatitis C screening (usually before blood transfusion) and hepatitis B immunization schedules (Wang et al., 2014), much remains to be done to meet the requirements for hepatitis elimination.

At present, the domestic research on viral hepatitis mainly focuses on its etiology, clinical features, epidemiology, and prevention and control policies, while few studies have been conducted on its temporal and spatial transmission. Nevertheless, relevant research results show that the spread of infectious diseases such as viral hepatitis and HIV is related to spatial factors (Busgeeth and Rivett, 2004; Rosenberg et al., 2018; Clipman et al., 2021). Ren et al. (Ren et al., 2022) have analyzed the distribution of HIV in Luzhou using Bayesian spatiotemporal model. Tian et al. (Tian et al., 2018) have used spatiotemporal analysis to study the impact of urbanization on hantavirus. Meanwhile, socioeconomics, income, education, occupation, and blood transfusion are all closely related to hepatitis B and C (Akbar et al., 1997; Salemi et al., 2017; Ahn et al., 2018). Additionally, accurate data is an important prerequisite for sound public health and health care policies and guidelines, allowing the health burden to drive resource allocation decisions and disseminating accurate information to health professionals, patients and the public. Therefore, this study used Bayesian spatiotemporal hierarchical model to analyze the influence of socioeconomic factors on the spatiotemporal distribution of hepatitis B and C in China from 2010 to 2018, and revealed provincial cold and hot spots in the time dimension. The posterior distribution was used to map the disease risk of hepatitis B and C, which provided new insights for the precise prevention and control of hepatitis B and C.

## 2 Methods

### 2.1 Ethics statement

This study has been approved by the ethics committee of Nanjing Bioengineering (Gene) Technology Center for Medicines (No:2021BY07). Patient consent was not required because no patients’ individual information was included in this study and population data were collected from the public database of China.

### 2.2 Data Sources

Annual data of hepatitis B and C cases for the period from 2010 to 2018 were obtained from the Chinese Center for Disease Control and Prevention (https://www.phsciencedata.cn/Share/). The case definition is based on the unified diagnostic criteria formulated by the Chinese Ministry of Health (MOH). The following demographic information used in the Bayesian space-time hierarchy model were acquired from the Chinese economic Statistical Year book (http://www.stats.gov.cn/): (1) population by region at the end of the year; (2) the proportion of illiterate population in the population aged 15 and above (%); (3) per capita gross domestic product (GDP) (the GDP divided by the population of the region); (4) road mileage by region (kilometer); (5) the urbanization rate (which is divided by the urban resident population); (6) the number of hygienic personnel per 1000 people; (7) beds in medical and health institutions per 1000 people; (8) and population density (the number of permanent residents divided by the total area of the province). The data and code used in this article are uploaded to the sharing platform Github: https://github.com/ykjjqian/BSTHM1/tree/master.

### 2.3 Bayesian space–time hierarchy model

In this study, we used the BSTHM (Richardson et al., 2004; Li et al., 2014a) with Poisson distribution to capture spatial and temporal heterogeneity of hepatitis B and C and quantity the association between the potential driving factors and the incidence of hepatitis B and C. In the model, we let  *y*_*it*_ , *n*_*it*_ and *u*_*it*_ represent the hepatitis B or C cases in province *i*(*i*=1,…,31) and year *t*(*t*=1,…,9) , the risk population, and the spatiotemporal risk of hepatitis B or C. *β*_1_  to *β*_8_ denote the regression coefficients of the potential driving factors.


$$y_{it}\sim Poisson(n_{it}u_{it})$$

$$\log\left(u_{it}\right)=\alpha +s_{i}+b_{0}t^{*}+v_{t}+\sum_{k=1}^{8}\beta_{k}x_{ik}+b_{1i}t^{*}+\epsilon_{it}$$

where α is the overall logarithm of hepatitis B or C risk in China over the nine years and *t* ^*^=*t*−4.5 (centering at the mid-observation period). The spatial term  *s*_*i*_ describes the spatial distribution of disease risk throughout the study period. The exp(*s*_*i*_) is the spatial disease risk, which is influenced by some related factors in the study period, such as economic conditions, and medical resources. The temporal term (*b*_0_ *t* ^*^+*v*_*t*_)  describes the overall temporal trend common to all provinces, and the overall temporal trend is specified as a linear trend (*b*_0_ *t* ^*^ ) with $v_{t}\sim N\left(0,\sigma_{v}^{2}\right)$ , which allows for nonlinearity of the overall trend pattern. The term *b*_1*i*_ *t* ^*^ allows each province to have its own trend and capture the departure extent from *b*_0_ for each region. A positive estimate represents a relatively rapid increase (or even decrease) of disease risk in that particular province over time. The last term $\epsilon_{it}\sim N\left(0,\sigma_{\epsilon }^{2}\right)$ (Gelman, 2006) is the Gaussian random noise variable and captures additional variability not yet explained by other model components. For such overdiscrete count data, this additional source of variability is mainly that the observed variability exceeds the variability that can be explained by the Poisson model(Johnson and Bowers, 2004). The prior distribution of the global spatial random effect term *s*_*i*_ is BYM model (Besag et al., 1991). The BYM model is a convolution of spatially structured random effects and spatial unstructured random effects, the latter following a Gaussian distribution. Meanwhile, the conditional autoregressive (CAR) prior with a space adjacency matrix *W*_31×31_ was used to impose spatial structure. If the country *i* and *j* shared a common border, then *W*_*ij*_=1 , otherwise, *W*_*ij*_=0 . *b*_1*i*_ *t* ^*^ has the same BYM prior as *s*_*i*_ . The CAR prior to the spatial random effect showed that neighboring provinces tended to have a similar overall risk of disease. Finally, a strict positive half-Gaussian prior *N*_+*∞*_(0,10) is assigned to all random effects standard deviations. *x*_*ik*_ is a covariate incorporated on the basis of the previous model that helps explain space/time patterns (Li et al., 2014a). K=8 represents the number of covariates, including illiteracy percentage aged 15 years and above, GDP per capita, regional road mileage, regional urbanization rate, number of hygienic personnel, beds in medical and health institutions, population density, and incidence rate of hepatitis B or hepatitis C. Assign the non-informational prior to the regression coefficient *β* . In Bayesian simulations, any interval that contains 95% of the posterior mass is a frequency confidence interval (CI), often called a credible interval (CRI), and sometimes called a Bayesian confidence interval. Generally, the 2.5th and 97.5th percentiles of the posterior sample are selected as the 95% CRI.

The provinces were classified into nine categories (3 risk categories × 3 trend categories) according to a two-stage classification rule (Richardson et al., 2004). In the first stage, a province was defined as a hotspot for posterior probability *P*(*exp*(*s*_*i*_)>1|*data*)∈[0.8,1] and as a coldspot for *P*(*exp*(*s*_*i*_)>1|*data*)∈[0,0.2] . If *P*(*exp*(*s*_*i*_)>1|*data*)∈(0.2,0.8) , the province is defined as neither hotspots nor coldspots. Hot and cold spots represent the province’s consistently above/below the average disease risk in China, which changes over time. In the second stage, according to the the local slopes *b*_1*i*_ , the provinces corresponding to each risk category in the first stage were classified into three trend patterns: level 1, the variation trend of the disease is faster than the overall trend, if *P*(*b*_1*i*_>0|*h*_*i*_,*data*)∈[0.8,1] ; level 2, the variation trend of the disease is slower than the overall trend, if *P*(*b*_1*i*_>0|*h*_*i*_,*data*)∈[0,0.2] ; level 3, the variation trend in the disease has no difference with the mean level, if *P*(*b*_1*i*_>0|*h*_*i*_,*data*)∈(0.2,0.8) . This is used to highlight provinces that have not yet become hot/cold spots but have a tendency to become hot spots. Richardson et al. (Richardson et al., 2004) have demonstrated that the probability cut-off used above to identify areas of very high/very low disease risk strikes a good balance between sensitivity (i.e., the ability to detect hot spots/cold spots when overall risk is indeed above/below the mean) and false-positive rates (i.e., the ratio of declared hot spots/cold spots where actual risk does not differ from the mean).

The whole BSTHM was performed in OpenBUGS (Richardson et al., 2004). The posterior distribution of model parameters was obtained by Markov chain Monte Carlo (MCMC) simulation. We ran two Markov chain Monte Carlo (MCMC) chains for 45,000 iterations and discarded the first 15,000 iterations as aging. The diagnosis of convergence of Bayesian estimates was assessed by the Brooks-Gelman-Rubin (BGR) ratio (Brooks and Gelman, 1998). The closer the ratio is to 1.0, the better the model converges (Li et al., 2014b). Of the total 236 parameters of the Bayesian space-time model, only 1.69% had a BGR ratio greater than 1.05.

## 3 Results

### 3.1 Demographic characteristics

**Figure 1:**
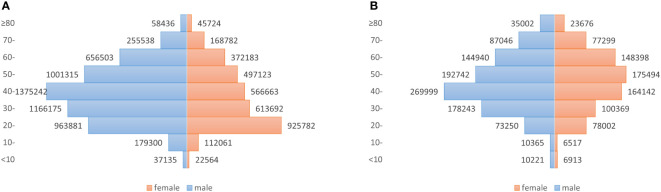
Number of newly diagnosed hepatitis B and hepatitis C cases for male and female patients in different age groups, in China, 2010–2018. **(A)** Hepatitis B, with a sex ratio of 1.71; **(B)** Hepatitis C, with a sex ratio of 1.28.

**Figure 2:**
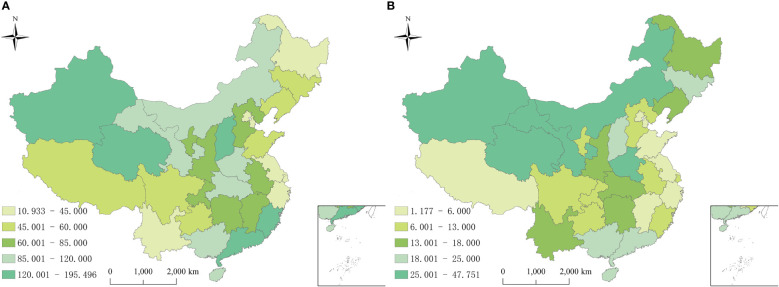
Annual average incidence rates of reported hepatitis B and C by province in China from 2010 to 2018. **(A)** Hepatitis B; **(B)** Hepatitis C.

From 2010 to 2018 in China, a total of 9018099 cases of hepatitis B and 1782618 cases of hepatitis C were reported in the study regions, with the average annual incidence of 75.93 and 15.52 per 100,000 people respectively. Of the total hepatitis B cases, 5693525 cases were males and 3324574 cases were females, with a sex ratio of 1.71. 1001808 cases of hepatitis C were males and 780810 cases were females, with the sex ratio of 1.28. All age groups were susceptible, and 90.25% (8138559/9018099) and 85.58% (1525579/1782618) of hepatitis B and C cases occurred in aged 20-60, respectively (**Figure 1**). Farmers were the majority group of hepatitis B and C, accounting for 53.79% (4312756/8018114) and 48.33% (755510/1563243), respectively (**Tables 1**, **2**). Geographically, Qinghai had the highest average incidence (195.50 cases per 100000 population) of hepatitis B from 2010 to 2018, 17.89 times higher than that in Beijing (10.93 cases per 100000 population), which had the lowest average incidence rate of hepatitis B. Xinjiang had the highest average incidence (47.75 cases per 100000 population) of hepatitis C, 40.47 times higher than that in Tibet(1.18 cases per 100000 population), which had the lowest average incidence rate of hepatitis C. **Figure 2** showed the incidence change in hepatitis B and hepatitis C in China from 2010 to 2018, respectively.

**Table 1:** Demographic characteristics of hepatitis B cases in mainland China, 2010–2018.

Hepatitis B group	2010	2011	2012	2013	2014	2015	2016	2017	2018	Total
Gender										
Male	675781 (0.64)	685544 (0.63)	679325 (0.62)	602913 (0.63)	587062 (0.63)	592930 (0.63)	597432 (0.63)	636961 (0.64)	635577 (0.64)	5693525 (0.63)
Female	384801 (0.36)	407791 (0.37)	407761 (0.38)	360061 (0.37)	348640 (0.37)	341285 (0.37)	344836 (0.37)	364991 (0.36)	364408 (0.36)	3324574 (0.37)
Sex ratio	1.76	1.68	1.67	1.67	1.68	1.74	1.73	1.75	1.74	1.71
Age										
0~	9931 (0.01)	8561 (0.01)	7695 (0.01)	6348 (0.01)	5901 (0.01)	5671 (0.01)	5060 (0.01)	5316 (0.01)	5216 (0.01)	59699 (0.01)
10~	62007 (0.06)	49891 (0.05)	41335 (0.04)	31809 (0.03)	26374 (0.03)	23253 (0.02)	20323 (0.02)	19380 (0.02)	16989 (0.02)	291361 (0.03)
20~	258060 (0.24)	270762 (0.25)	258664 (0.24)	216558 (0.22)	201159 (0.21)	186002 (0.20)	176813 (0.19)	170311 (0.17)	151334 (0.15)	1889663 (0.21)
30~	224181 (0.21)	223587 (0.20)	215381 (0.20)	187848 (0.20)	178316 (0.19)	175911 (0.17)	180524 (0.19)	195952 (0.20)	198167 (0.20)	1779867 (0.20)
40~	215394 (0.20)	231177 (0.21)	233778 (0.22)	209722 (0.22)	204620 (0.22)	205756 (0.22)	205636 (0.22)	219598 (0.22)	216224 (0.22)	1941905 (0.22)
50~	151925 (0.14)	156906 (0.14)	162973 (0.15)	154100 (0.16)	156574 (0.17)	163683 (0.18)	170883 (0.18)	186043 (0.19)	195351 (0.20)	1498438 (0.17)
60~	89984 (0.08)	99456 (0.09)	109268 (0.10)	103616 (0.11)	107904 (0.12)	115762 (0.12)	122621 (0.13)	136504 (0.14)	143571 (0.14)	1028686 (0.11)
70~	40364 (0.04)	43392 (0.04)	47251 (0.04)	42773 (0.04)	43762 (0.05)	46194 (0.05)	48182 (0.05)	54422 (0.05)	57980 (0.06)	424320 (0.05)
80~	8736 (0.01)	9603 (0.01)	10741 (0.01)	10200 (0.01)	11092 (0.01)	11983 (0.01)	12226 (0.01)	14426 (0.01)	15153 (0.02)	104160 (0.01)
Occupation										
Farmers	513975 (0.48)	573538 (0.52)	581847 (0.54)	529647 (0.55)	513294 (0.55)	517927 (0.55)	524154 (0.56)	558374 (0.56)	–	4312756 (0.54)
Housework and unemployment	98654 (0.09)	117979 (0.11)	122827 (0.11)	125158 (0.13)	128704 (0.14)	132906 (0.14)	136909 (0.15)	149163 (0.15)	–	1012300 (0.13)
Worker	111147 (0.10)	76051 (0.07)	65956 (0.06)	57631 (0.06)	55909 (0.06)	53187 (0.06)	51501 (0.05)	53000 (0.05)	–	524382 (0.07)
Retiree	45571 (0.04)	47430 (0.04)	49247 (0.05)	45044 (0.05)	43429 (0.05)	44479 (0.05)	47089 (0.05)	48406 (0.05)	–	370695 (0.12)
Business service personnel	26762 (0.03)	29260 (0.03)	30691 (0.03)	32525 (0.03)	33597 (0.04)	34013 (0.04)	37054 (0.04)	43015 (0.04)	–	266917 (0.03)
Cadres and staff	44032 (0.04)	36427 (0.03)	32845 (0.03)	27877 (0.03)	25026 (0.03)	23639 (0.03)	24727 (0.03)	24829 (0.02)	–	239402 (0.03)
Others	31203 (0.21)	34668 (0.19)	37589 (0.19)	28459 (0.15)	27997 (0.15)	27552 (0.14)	26064 (0.13)	25774 (0.12)	–	239306 (0.16)

**Table 2:** Demographic characteristics of hepatitis C cases in mainland China, 2010–2018.

Hepatitis C group	2010	2011	2012	2013	2014	2015	2016	2017	2018	Total
Gender										
Male	88041 (0.58)	99179 (0.57)	113315 (0.56)	111954 (0.55)	112162 (0.55)	115616 (0.56)	116092 (0.56)	120731 (0.56)	124718 (0.57)	1001808 (0.56)
Female	64998 (0.42)	74693 (0.43)	88307 (0.44)	91201 (0.45)	90641 (0.45)	92281 (0.44)	90740 (0.44)	93292 (0.44)	94657 (0.43)	780810 (0.44)
Sex ratio	1.35	1.33	1.28	1.23	1.24	1.25	1.28	1.29	1.32	1.28
Age										
0~	2006 (0.01)	2170 (0.01)	2647 (0.01)	2266 (0.01)	2001 (0.01)	1860 (0.01)	1666 (0.01)	1408 (0.01)	1110 (0.01)	17134 (0.01)
10~	2749 (0.02)	2814 (0.02)	2961 (0.01)	2361 (0.01)	1660 (0.01)	1261 (0.01)	1103 (0.01)	1069 (0.00)	904 (0.00)	16882 (0.01)
20~	15988 (0.10)	17726 (0.10)	18932 (0.09)	19234 (0.09)	18297 (0.09)	17234 (0.08)	16084 (0.08)	14872 (0.07)	12885 (0.06)	151252 (0.08)
30~	31144 (0.20)	33725 (0.19)	35830 (0.18)	33944 (0.17)	31913 (0.16)	30065 (0.14)	28646 (0.14)	27608 (0.13)	25737 (0.12)	278612 (0.17)
40~	34093 (0.22)	42194 (0.24)	50187 (0.25)	50854 (0.25)	51080 (0.25)	51685 (0.25)	50800 (0.25)	51489 (0.24)	51759 (0.24)	434141 (0.24)
50~	26368 (0.17)	30402 (0.17)	37674 (0.19)	39791 (0.20)	41811 (0.21)	45056 (0.22)	45766 (0.22)	48382 (0.23)	52986 (0.24)	368236 (0.21)
60~	20626 (0.13)	23766 (0.14)	29032 (0.14)	30668 (0.15)	32586 (0.16)	35740 (0.17)	36920 (0.18)	40419 (0.19)	43581 (0.20)	293338 (0.16)
70~	14981 (0.10)	15777 (0.09)	18153 (0.09)	17746 (0.09)	17341 (0.09)	18329 (0.09)	18854 (0.09)	20948 (0.10)	22216 (0.10)	164345 (0.09)
80~	5084 (0.03)	5298 (0.03)	6206 (0.03)	6291 (0.03)	6114 (0.03)	6667 (0.03)	6993 (0.03)	7828 (0.04)	8197 (0.04)	58678 (0.03)
Occupation										
Farmers	58856 (0.38)	74173 (0.43)	93015 (0.46)	100110 (0.49)	101184 (0.50)	106432 (0.51)	107351 (0.52)	114389 (0.53)	–	755510 (0.48)
Housework and unemployment	21183 (0.14)	26463 (0.15)	30241 (0.15)	34444 (0.17)	34549 (0.17)	35243 (0.17)	35584 (0.17)	36821 (0.17)	–	254528 (0.16)
Worker	14563 (0.10)	10999 (0.06)	11310 (0.06)	10942 (0.05)	10487 (0.05)	9991 (0.05)	9065 (0.04)	8769 (0.04)	–	86126 (0.06)
Retiree	18127 (0.11)	18635 (0.10)	19939 (0.09)	19255 (0.09)	18725 (0.09)	19315 (0.09)	19424 (0.09)	18758 (0.09)	–	152178 (0.10)
Business service personnel	2830 (0.02)	3425 (0.02)	4066 (0.02)	4803 (0.02)	5126 (0.03)	4851 (0.02)	5238 (0.03)	5647 (0.03)	–	35986 (0.02)
Cadres and staff	6277 (0.04)	5509 (0.03)	5462 (0.03)	5142 (0.03)	4735 (0.02)	4513 (0.02)	4106 (0.02)	3865 (0.02)	–	39609 (0.03)
Others	220441 (0.20)	212650 (0.20)	203673 (0.19)	145092 (0.14)	135743 (0.14)	128064 (0.13)	120834 (0.13)	125165 (0.12)		1291662 (0.15)

### 3.2 Temporal trend

**Figure 3:**
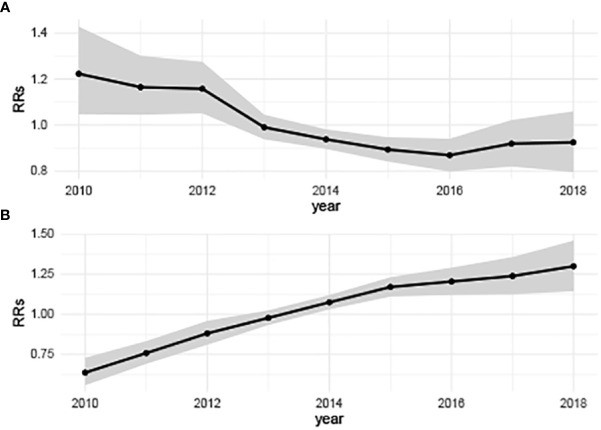
Annually temporal relative risks of hepatitis B and hepatitis C in China from 2010 to 2018. **(A)** Hepatitis B, showing a slow downward trend; **(B)** Hepatitis C, showing a significant upward trend. (*b*_0_ *t* ^*^+*v*_*t*_)  describes the overall time trend common to all counties with $v_{t}\sim N\left(0,\sigma_{v}^{2}\right)$ .

On the time perspective, the overall temporal variation of hepatitis B showed a slow downward trend, while the relative risks (RRs) of hepatitis C showed an overall upward trend from 2010 to 2018 (**Figure 3**). The highest disease risk of hepatitis B (RRs: 1.22, 95%CRI: 1.05-1.43) occurred in 2010 and the lowest disease risk (RRs: 0.87, 95%CRI: 0.80-0.94) occurred in 2016. In 2010, the hepatitis C temporal RRs (0.64, 95%CRI: 0.56-0.73) was the lowest, while in 2018, the temporal RRs of hepatitis C (1.30, 95%CRI:1.14-1.46) was the highest.

### 3.3 Spatial heterogeneity

**Figure 4:**
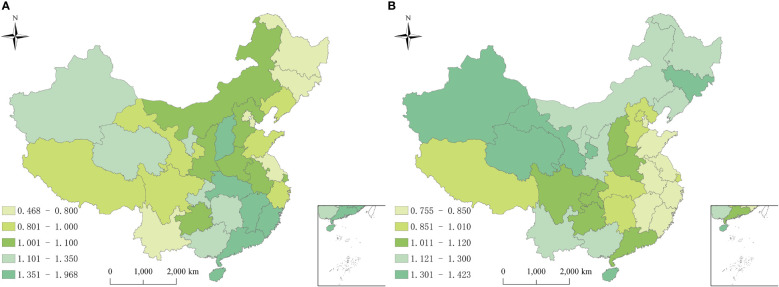
Spatial relative risks of hepatitis B and hepatitis C in China from 2010 to 2018. **(A)** Hepatitis B, high risk areas were mainly distributed in the southeast and northwest regions; **(B)** Hepatitis B, high risk areas were mainly distributed in the north. The *exp*(*s*_*i*_) is the spatial risk of this disease, which is influenced by some related factors in the study period, such as economic conditions, local prevention and control policies, and medical resources.

Geographically, the spatial relative risks (RRs) of hepatitis B and C calculated using the BHSTM were different substantially, indicating significant heterogeneity in both hepatitis B and C incidence risk in the study region. **Figure 4** showed the spatial RRs of hepatitis B and C at the province level from 2010 to 2018. The provinces with a higher spatial risk of hepatitis B were mainly distributed in the southeast and northwest regions, while the risk of hepatitis C was relatively higher in northern China.

**Figure 5:**
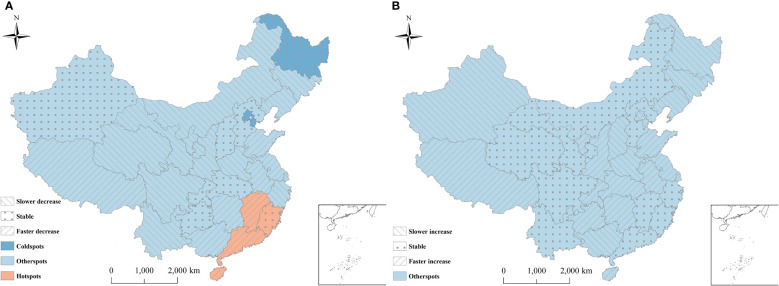
Distribution of hot and cold spots of hepatitis B and hepatitis C in China. **(A)** Hepatitis B; **(B)** Hepatitis C. The provinces were classified into nine categories (3 risk categories × 3 trend categories). 3 risk categories: hotspots: *P*(*exp*(*s*_*i*_)>1|*data*)∈[0.8, 1] , representing high-risk area; coldspots: *P*(*exp*(*s*_*i*_)>1|*data*)∈[0,0.2] , representing low-risk area; neither hotspots nor coldspots: *P*(*exp*(*s*_*i*_)>1|*data*)∈(0.2,0.8) . 3 trend categories: level 1, the variation trend of the disease is faster than the overall trend, if *P*(*b*_1*i*_>0|*h*_*i*_,*data*)∈[0.8,1] ; level 2, the variation trend of the disease is slower than the overall trend, if *P*(*b*_1*i*_>0|*h*_*i*_,*data*)∈[0,0.2] ; level 3, the variation trend in the disease has no difference with the mean level, if *P*(*b*_1*i*_>0|*h*_*i*_,*data*)∈(0.2,0.8).

**Figure 5** showed the spatial patterns of hot and cold spots of hepatitis B and C in 2010-2018. For hepatitis B, 3/31 (9.68%) and 4/31 (12.90%) provinces were identified as cold spots and hot spots, respectively. The remaining 24/31(77.42%) provinces were defined as neither cold spots nor hot spots. The provinces in hotspots with a high spatial RRs value were located mainly in the southeast (Jiangxi, Fujian, Guangdong, and Hainan). Thus, the hepatitis B risk was relatively high in these provinces. The provinces in cold spots with a low spatial RRs value were located mainly in southwest (Heilongjiang, Tianjin, and Beijing), indicating a low level of hepatitis B. For hepatitis C, all provinces were defined as neither cold spots nor hot spots (**Figure 5**).

For hepatitis B, among the four hot spots, 75% (Hainan, Guangdong and Jiangxi) of all hotspots showed a faster temporal decreasing trend than the overall decreasing trend. Consequently, these regions might become lower risk regions or even non-hotspots in the future. Meanwhile, 25%(Fujian) of the hotspots showed the same trends as the overall trend, which indicated that these regions would still be hot areas over time (**Figure 5**). Therefore, the public health sector should focus on these provinces. Among the three cold spots, the provinces showed the same trends as the overall trend, indicating that these regions would likely remain cold areas, with a low risk (**Figure 5**).

Among the remaining twenty-four provinces of neither hot spots nor cold spots, approximately 45.83% (Inner Mongolia, Liaoning, Jilin, Ningxia, Gansu, Qinghai, Sichuan, Shaanxi, Henan, Zhejiang, and Yunnan) showed a slower decreasing trend than the overall decreasing trend, indicating that these regions might become higher risk regions or change into hot spots over time. Meanwhile, 29.17% (Tibet, Guangxi, Anhui, Jiangsu, Shandong, Shanghai, and Hunan) showed a faster decreasing trend than the overall trend, indicating that these regions might become lower risk areas or even cold spots over time. The remaining six provinces were consistent with the trend, with the current risk level over time (**Figure 5**).

For hepatitis C, among all provinces, 41.94% (Tibet, Guizhou, Hunan, Chongqing, Shandong, Shaanxi, Ningxia, Hubei, Anhui, Jiangsu, Shanghai, Hainan, and Tianjin) of neither hot spots nor cold spots exhibited a faster increasing trend than the overall increasing trend, indicating that these regions might become higher risk regions or even become hot spots in the future. 33.33% (Xinjiang, Heilongjiang, Jilin, Liaoning, Beijing, Shanxi, Henan, and Guangxi) of neither hot spots nor cold spots showed a slower upwards than the overall increasing trend. Consequently, the risk in these provinces would likely be lower than the overall risk, and they might become cold spots over time. The remaining provinces were consistent with the overall trend. Thus, the current risk level in these provinces will remain constant in the future (**Figure 5**).

### 3.4 Risk factor detection

We used a Bayesian space-time hierarchy mode**l** to analyze the impact of the factors, such as urbanization rate, per capita GDP, illiterate rate, road mileage, hygienic personnel, beds, and density, on hepatitis B and C. The results found that the increase of the incidence rate of hepatitis C, and illiteracy rate increased the RRs of having hepatitis B (**Table 3**). For hepatitis C, the increase of illiteracy rate, and per capita GDP were protective factors, while the increase of incidence rate of hepatitis B increased the RRs of having hepatitis C (**Table 4**).

**Table 3:** Quantified posterior mean values and relative risks (RRs) of potential driving factors of hepatitis B in China from 2010 to 2018.

Variable	Posterior mean(95%CRI)	RRs (95%CRI)
Per capita GDP	-0.0004(-0.0011, 0.0003)	1.0000(1.0000,1.0000)
Urbanization rate (%)	1.0900(-0.8540, 3.1520)	1.0110(0.9915,1.0320)
Incidence rate of hepatitis C (%)	2.7410(1.9610, 3.4800)	1.0280(1.0200,1.0350)
Illiteracy rate (%)	2.5430(0.9444, 4.2530)	1.0260(1.0090,1.0430)

**Table 4:** Quantified posterior mean values and relative risks (RRs) of potential driving factors of hepatitis C in China from 2010 to 2018.

Variable	Posterior mean(95%CRI)	RRs (95%CRI)
Per capita GDP	-0.0008(-0.0015, -0.0003)	1.0000(1.0000,1.0000)
Urbanization rate (%)	0.4989(-0.7851, 2.4170)	1.0005(0.9922,1.0240)
Incidence rate of hepatitis B (%)	0.3866(0.2699, 0.5132)	1.0040(1.0030,1.0050)
Illiteracy rate (%)	-3.1830(-4.7090, -1.6450)	0.9687(0.9540,0.9837)

An increase of 1 yuan in per capita GDP was related to a decrease of 0.0008% (-0.0015, -0.0003) in the risk of hepatitis C (RRs: 1.0000; 95%CRI: 1.0000-1.0000). Every 1% increase in illiteracy rate was related to a 2.5430% (0.9444, 4.2530) increase in hepatitis B risk, with a corresponding RRs of 1.0264(95%CRI: 1.0090-1.0430). By contrast, a 1% increase in illiteracy rate was related to a 3.1830% (-4.7090, -1.6450) decreases in hepatitis C risk, with a corresponding RRs of 0.9687(95%CRI: 0.9540-0.9837) (**Tables 3**, **4**).

Increased prevalence of hepatitis B and hepatitis C can increase the risk of hepatitis C and hepatitis B, respectively. A 1% increase in the incidence of hepatitis C was related to an increase of 2.7410% (95%CRI: 1.9610-3.4800) in the risk of hepatitis (RRs: 1.0280; 95%CRI: 1.0200,1.0350). Meanwhile, every 1% increase in the incidence of hepatitis B was related to a 0.3866% (0.2699, 0.5132) increase in hepatitis C risk, with a corresponding RRs of 1.0040(95%CRI: 1.0030-1.0050). The influence of the remaining factors on the risk of acquiring hepatitis B or hepatitis C infection was not significant (**Tables 3**, **4**).

## 4 Discussion

In this study, we used Bayesian spatiotemporal hierarchy models to study the spatiotemporal heterogeneity of hepatitis B and C in China and measured the potential impact of socioeconomic factors on hepatitis B and hepatitis C in China, based on the national disease surveillance dataset from 2010 to 2018 of the Chinese Center for Disease Control and Prevention. BSTHM embeds spatiotemporal information, prior distribution and spatiotemporal correlation factors, which solves the estimation bias caused by spatial structure and makes the estimation more stable and reliable (Best et al., 2005). The results showed significant spatial and temporal heterogeneity in the risk of hepatitis B and C. Over time, the risk of hepatitis B had generally shown a slow downward trend, while the risk of hepatitis C had been on the rise. Spatially, the high-risk areas of hepatitis B, were mainly distributed in the southeast and northwest regions, while the high-risk areas of hepatitis C were mainly distributed in the northern regions. In addition, for hepatitis B, illiteracy, and hepatitis C prevalence were the main contributing factors, while GDP per capita, illiteracy rate, and hepatitis B prevalence were the main contributing factors to hepatitis C.

The spatial distribution of viral hepatitis was uneven, indicating that socioeconomic conditions were strongly associated with viral hepatitis risk. For example, increased prevalence of hepatitis B and hepatitis C can increase the risk of hepatitis C and hepatitis B, respectively. The illiteracy rate of people aged 15 and above represents the local education level to some extent, and the illiteracy rate in Fujian showed an upward trend, which partly explained the high risk in Fujian. On the other hand, HBV detection was removed from routine health check-ups for new employees and students from 2010 due to population discrimination against people with hepatitis B (Cooke et al., 2019), which would also affect the diagnosis of hepatitis B and may be an important factor in the decline in the prevalence of hepatitis B. For hepatitis C, an increase of 1 yuan in per capita GDP was related to a decrease of 0.0008% in the risk of hepatitis B. High risk areas of hepatitis C were mainly distributed in the north. The per capita GDP in the north was lower than that in the south, while Beijing and Tianjin in the north were in the forefront of the country. With their high level of culture and medical care and complete infrastructure, they had a low risk of hepatitis C. Therefore, per capita GDP was a protective factor against hepatitis C. The results of this research also showed that the improvement of education level (the reduction of illiteracy rate) had increased the RRs value of hepatitis C, which might be attributed to but not limited to the following reasons: on the one hand, since 2009, the Center for Sexual AIDS Prevention and Control of the Chinese Center for Disease Control and Prevention has carried out comprehensive prevention and treatment of hepatitis C, and in 2012, the Office of Hepatitis C and STD Prevention and Control was established to explore the “Chinese experience and model” of eliminating hepatitis C, and do a good job in hepatitis C publicity and education and comprehensive intervention. The prevention and control level of hepatitis C by the population and relevant staff had been improved, and the detection rate of hepatitis C had been improved. On the other hand, with the improvement of literacy level, people recognized that hepatitis C was preventable and curable, national medical insurance and other policy measures reduce public fear of hepatitis C and discrimination against patients, improve self-protection and positive medical awareness (Li et al., 2022). At present, China’s viral hepatitis control system is relatively fragmented, and at the same time, the funds clearly allocated to hepatitis C are relatively small (Chen et al., 2020), so the process of preventing and treating hepatitis C needs to enhance the top-level design to make up for the lack of financial and personnel support to a certain extent.

In addition, the research results showed that the increased prevalence of hepatitis B and hepatitis C would increase the risk of hepatitis C and hepatitis B, respectively, which to some extent indicated that people with hepatitis B or one of hepatitis C were often high-risk groups for another type of hepatitis. Relevant research showed that the incidence of co-infection of hepatitis B and hepatitis C was between 1% and 15%, while the presence of unidentified occult HBV infection might lead to the underestimated incidence (Senturk et al., 2008; Pol et al., 2017). Compared with single infection, HBV/HCV co infection will increase the severity of liver disease (Mavilia and Wu, 2018). In addition, some studies had revealed that hepatitis C treatment can reactivate hepatitis B (Blackard and Sherman, 2018; Ma and Feld, 2018). Therefore, surveillance of people who already have hepatitis C or B should be strengthened to reduce co-infection.

The study had some limitations. We used provincial data to explore population-level associations, which may inevitably lead to ecological fallacies (Jelinski and Wu, 1996), but this does not affect long-term trends in hepatitis B and C. Furthermore, the indicators used in the model are all macro-control statistics, but the elements affecting hepatitis are complex and diverse, so factors other than those considered in this study may bring some uncertainty to hepatitis B and C. Finally, there may be a delay or later between the reported number of hepatitis infections and the exact number of hepatitis infections, resulting in differences in RRs.

In short, the burden of hepatitis B and C in China remains high, and prevention and treatment faces many challenges (Wang et al., 2017), including economic development, education level, allocation of prevention and control resources, etc., which are important factors affecting hepatitis. In order to promote the prevention and control of the prevalence of hepatitis B and hepatitis C in China, we put forward the following suggestions: Firstly, China should set up special institutions to rationally allocate resources for hepatitis prevention and control (strengthen the prevention and control of hepatitis B in the southeast and northwest and hepatitis C in the north), and coordinate the cooperation among public health institutions, medical care providers,and communities to ensure the effective use of resources and expertise. Secondly, stigma and discrimination related to hepatitis B and hepatitis C are also a serious obstacle. Medical professionals should actively participate in and provide relevant publicity activities to improve public awareness and eliminate discrimination, while respecting the privacy of infected persons (Buti et al., 2022). Finally, scientific diagnostic criteria and screening technology (especially mixed infection) and advanced modeling technology are crucial for monitoring and eliminating hepatitis. Therefore, we can use GISAID, Github, and other data sharing platforms to manage, share and analyze data and promote the optimization of public prevention and control measures.

## Data availability statement

The original contributions presented in the study are included in the article/supplementary material. Further inquiries can be directed to the corresponding authors.

## Author contributions

Funding acquisition: CHW, WT. Data accept: JQ, LA, MY, YL. Data analysis: JQ, MY, NY, CCW. Project administration: YZ, CHW, CZ. Supervision: CHW, PH. Writing-original draft: JQ, WT, MY. Writing-review and editing: CHW, JQ, WT. All authors contributed to the article and approved the submitted version.
